# High efficient removal of lead(II) and cadmium(II) ions from multi-component aqueous solutions using polyacrylic acid acrylonitrile talc nanocomposite

**DOI:** 10.1007/s11356-022-21023-1

**Published:** 2022-05-26

**Authors:** Mohamed Ragab Abass, Wafaa Mohamed El-Kenany, Eman Hassan EL-Masry

**Affiliations:** Hot Laboratories and Waste Management Center, Egyptian Atomic Energy Authority, Cairo, 13759 Egypt

**Keywords:** Gamma-irradiation, Polymerization, Talc, Distribution coefficients, Thermodynamic, Column

## Abstract

This study is interested in the removal of Pb(II), Cd(II), Co(II), Zn(II), and Sr(II) onto polyacrylic acid acrylonitrile talc P(AA-AN)-talc nanocomposite. P(AA-AN)-talc was fabricated using γ-irradiation-initiated polymerization at 50 kGy. Different analytical tools were used to investigate the functional groups, morphology, particle size, and structure of this composite. The ability of P(AA-AN)-talc to capture (Pb^2+^, Cd^2+^, Co^2+^, Zn^2+^, and Sr^2+^) as multi-component aqueous solutions was performed by a batch method. Saturation capacity and the effect of (agitating time, pH, initial metal concentrations, and temperature) were investigated. The distribution coefficients at different pHs have order: Pb^2+^  > Cd^2+^  > Co^2+^  > Zn^2+^  > Sr^2+^. The saturation capacity decreases by increasing heating temperatures. Reaction kinetic obeys the pseudo-second-order model. Sorption isotherms are more relevant to a Langmuir isotherm, and the monolayer sorption capacity is closed to saturation capacity. Thermodynamic parameters (∆G˚, ∆H˚, and ∆S˚) were endothermic and spontaneous. P(AA-AN)-talc is used for loading and recovery of studied cations in the column system. The study confirms that P(AA-AN)-talc is a promised composite for the sorption of the studied ions from aqueous solutions and should be considered as potential material for decontaminating these ions.

**Graphical abstract:**

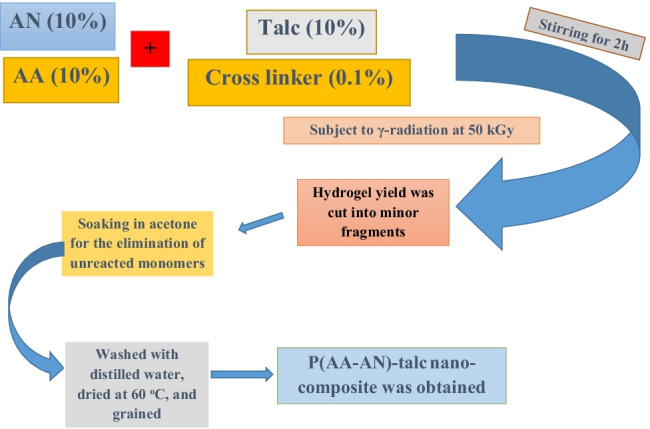

## Introduction

Radioactive waste is obtained through a wide scale of operations in research facilities, recycling plants, power plants, diagnostic medicine, and isotope manufacturing (Nilchi et al. 2007; Hamed et al. 2016b). The stream of radioactive waste from nuclear facilities includes low, intermediate, and highly radioactive wastes and also includes emitting isotopes. Furthermore, these streams could contain different hazardous elements from decontamination procedures. These wastes must be treated to decrease dangerous element concentrations to levels suitable for discharge into the environment (Nilchi et al. 2007). Recently, the contaminants captured from wastewater have become a major challenge, as their significance is extending with increasing industrial activities (Hamoud et al. 2014). Various technologies can be used to remove pollutants as, for example, chemical precipitation (Ahn et al. 2020), physical treatment like solvent extraction (Brockmeyer et al. 2015; Hurtado-Bermúdez et al. 2018), adsorption (Abass et al. 2021a, b), biosorption (Gupta et al. 2019), membrane (Mehta et al. 2020), and ion exchange (El-Dessouky et al. 2018). Because of the high toxicity of Pb(II) and their widespread presence in the area, lead ions have a particular interest. It is an industrial contaminant, which arrives in the ecosystem through the soil, air, and/or water (Arbabi et al. 2015). Cadmium is common for electroplating, nuclear reactors, and certain industrial paints (Aglan et al. 2019). Cd(II) has several isotopes along with ^109^Cd; its half-life is 1.27 years and is used as rods for controlling and shielding the absorption of neutrons in reactors with supplementary components (Aglan et al. 2019). Co(II) is used in nuclear, medical, enamel, semiconductor industries, and electricity galvanizing. Co(II) can cause adversative effects on health like asthma, heart damage, and destroying the thyroid gland and liver (Kulkarni 2016). Zinc is obtained through effluents discharged from industries, like pigments, electroplating, battery manufacturing, metallurgy, and municipal wastewater management plants. Zn(II) is a toxic ion and can cause damage to human life by bioaccumulating in the food chain (Zhang et al. 2017). Strontium radionuclide is formed by ^89^Sr (*t*_0.5_ = 51d) and ^90^Sr (*t*_0.5_ = 29y) and is produced not only from nuclear plants as fission radionuclides but also from nuclear fuel rods in the pre-treatment phase (Ali et al. 2020). The retention of Cd(II), Co(II), Pb(II), Zn(II), and Sr(II) from liquid and radioactive waste solutions has been achieved by many researchers (Wang et al. 2010; Dubey et al. 2014; Kang et al. 2016; Zhang et al. 2017; Aglan et al. 2019; Pyrzynska 2019).

Talc ore is a naturally occurring, low-cost, and small particle size material. Talc ore has a platy structure that consists of three layers, including the Mg-OH layer sandwiched among two (SiO_3_)^2−^ layers. Adjacent layers are linked by van der Waals forces. Talc powder surface has a huge number of Si–O-Si, Mg-O, OH, and O-Si–O bonds which will coordinate with transition elements in H_2_O or soil to capture them on the talc powder surface (Thi Huong et al. 2020). Talc is commonly used as a filler, coating, and dusting agent in paints, plastics, lubricants, pharmaceuticals, papers, cosmetics, and ceramics manufacture. There are few studies on the adsorption characteristics of heavy metal ions in talc (Andrić et al. 2014; Kalantari et al. 2014). Modified talcum powder using 50% HNO_3_ acid was used for sorption of methylene blue from wastewater (Wenlei et al. 2014). Fe_3_O_4_/Talc nanocomposite was fabricated by the coprecipitation-ultrasonication technique and used for the capture of Cr(IV) from aqueous solutions (Thi Huong et al. 2020). Fe_3_O_4_/talc nanocomposite was used for the removal of Cu(II), Ni(II), and Pb(II) ions from aqueous solutions (Kalantari et al. 2014).

Polymeric resins have some advantages like high capacity, non-toxic, high chemical stability, and low production cost, but they have some disadvantages such as low selectivity toward hazardous ions, ability to swilling, low radiation, and thermal stability. The sorption efficiency of the talc can be improved by adding polymeric resins. The impregnation of talc ore to organic polymer is more useful due to creating new materials having higher sorption capacity, fast adsorption rate, and selectivity for target elements, as well as reducing some disadvantages present in polymeric resins. The novelty of this study is the impregnation of talc inside polymeric resin layers using the gamma radiation technique as a new nanocomposite with high sorption performance for some metal ions from aqueous solutions, in addition to the need to develop and use new economic materials that have specifications suitable for working in radioactive waste treatment conditions.

In the present work, the fabrication and characterization of P(AA-AN)-talc nanocomposite using gamma radiation at 50 kGy were achieved. The sorption behavior of Cd(II), Co(II), Pb(II), Zn(II), and Sr(II) onto P(AA-AN)-talc nanocomposite is discussed. Chromatographic separations of binary and multi-component systems were applied on P(AA-AN)-talc nanocomposite.

## Experimental

### Materials

All salts and reagents in this work have an analytical grade and are used without further purification. CoCl_2_.6H_2_O, SrCl_2_, PbNO_3_, ZnCl_2_, CdCl_2_, acrylic acid (AA), and acrylonitrile (AN) from Alpha Chemika, India; HCl and HNO_3_ from Merck, Germany; and sodium hydroxide from El-Nasr Co., Egypt. Double distilled water (DDW) was used for all experiments and nanocomposite fabrication.

### Gamma cell

Co^60^ γ-cell source of class MC-20 (Russia) was operated as radiation of polymerization process at the Cyclotron factory, Egypt. The rate of the dose was ~ 473.35 Gy/h.

### Fabrication of P(AA-AN)-talc

At 303 ± 2 K and constant agitation, 10% (AA and AN) solutions were added dropwise to 10% talc solution, and 0.1% N, N-methylene-bisacrylamide (cross-linker) was distributed for the production of P(AA-AN)-talc with volumetric ratios (AA:AN: talc) equal unity. The solution remained agitated for 2 h to overcome complete homogeneity then subject to γ-irradiation at 50 kGy over a time of about 105 h. After radiation, the hydrogel was cut into small fragments, soaked in acetone to eliminate unreacted monomers, then washed with DDW, and dried at 333 ± 2 K. The solid was ground to powder and converted into the H^+^ form by mixing for 1 day with 0.1 M HNO_3_. The solid product was decanted and rinsed with DDW to eliminate the excess acid and dried at 333 ± 2 K.

### Instruments

The studied material was analyzed by Fourier transform infrared spectroscopy (FT-IR) (Alpha II Bruker, Germany) at 4000–400 cm^−1^. X-ray diffraction (XRD) was done (Shimadzu XD-D1, Japan). Differential thermal analysis (DTA) and thermogravimetric analysis (TGA) were done by a Shimadzu DTG-60 H. The surface morphology of the solid was recorded by scanning electron microscope (SEM) model Philips XL 30. The percent of elemental composition was detected by energy-dispersive X-ray (EDX) analysis. Transmission electron microscopy (TEM) and selected area electron diffraction (SAED) pictures were performed using JEM2100, Jeol. s.b, Japan. Atomic absorption spectrophotometer (Buck Scientific, VGP 210) was used to measure the concentrations of Cd(II)_,_ Co(II), Pb(II), Zn(II), and Sr(II).

### Chemical stability

The stability of P(AA-AN)-talc to different solvents was achieved by mixing 0.05 g of solid and 50 mL of H_2_O, HNO_3_, HCl, and NaOH in concentrations [1–6 M] in the desired solution with intermittent agitating for about three days at 298 ± 1 K. The filtrates were dried using IR lamps to dry, and then, the residue was tested gravimetrically by weight difference by a sensitive analytical balance(Abou-Mesalam et al. 2018).

### Point zero charge (PZC) determination

PZC for P(AA-AN)-talc was investigated by using a 10-mL bottle, and 0.1 g of solid was added to 10 mL of desired pH solutions. The pH was adjusted by 0.1 M (HCl and NH_3_) to find the different pHs of (2–12). The pH of the supernatant in each tube was represented as pH_i_. The samples were agitating for 24 h using a rotary agitator at 200 rpm. After settling, the pH of the supernatant in each tube was measured and represented as pH_f_. The PZC was determined from the plot of ΔpH versus pH_i_.

## Adsorption experiments

### Preliminary study

The experiment was done by shaking 0.05 g of two samples (talc and P(AA-AN)-talc) with 5 mL of Pb(II), Cd(II)_,_ Co(II), Zn(II), and Sr(II) (100 mg/L) with *V*/*m* = 100 mL/g in a shaker thermostat (Kottermann D-1362, Germany) at 300 ± 1 K. Sorbent and the sorbate solution were agitated in a shaker thermostat at 180 rmp and after sorption (24 h), and the samples were separated from the solution by centrifuge. Ion concentrations in the test solutions were detected before and after sorption by AAS. The uptake percentage is calculated by Eq. (1) (Khataee et al. 2013; Hassani et al. 2014; Hamed et al. 2019):

1 $$\%\mathrm{Uptake}\;\mathrm{of}\;\mathrm{Cd}^{2+},\mathrm{Co}^{2+},\mathrm{Pb}^{2+},\mathrm{Zn}^{2+},\;\mathrm{and}\;\mathrm{Sr}^{2+}\;\mathrm{=}\left(\frac{C_i-C_f}{C_i}\right)100$$

where *C*_*i*_ and *C*_*f*_ are the initial and final concentrations of sorbed metal ions in solution, respectively, and the data are tabulated in Table 1. These results reveal that the percent uptake was increased from about 68.2 to 96.9% for Pb(II), from about 61.8 to 91.1% for Cd(II), from about 42.2 to 48.9% for Co(II), from about 21.5 to 29.5% for Zn(II), and from about 8.2 to 11.8% for Sr(II); these data confirm that a great enhancement was noticed in the sorption of Pb(II), Cd(II), Co(II), Zn(II), and Sr(II) onto P(AA-AN)-talc, and this modified nanocomposite was used for further experimental work.

**Table 1:** The % uptake of Pb(II), Cd(II)_,_ Co(II), Zn(II), and Sr(II) sorbed onto talc and P(AA-AN)-talc at 300 ± 1 K

Samples	% Uptake				
	Pb(II)	Cd(II)	Co(II)	Zn(II)	Sr(II)
Talc	68.2	61.8	42.2	21.5	8.2
P(AA-AN)-talc	96.9	91.1	48.9	29.5	11.8

Batch sorption studies of the Pb(II), Cd(II)_,_ Co(II), Zn(II), and Sr(II) onto P(AA-AN)-talc in H^+^ form. The variation in sorption parameters such as pH (1–5), contact time (5–240 min), metal ion concentrations (50–600 mg/L), and temperatures (300, 313, and 333 K) is patterned to get the optimum condition for the sorption process.

The separation factor may be considered as the relative tendency of different ions to be adsorbed in an exchanger from solutions. It is used as a measure of the possibility of chromatographic separation and is also expressed as the ratio of the distribution coefficients of the ions to be separated. The distribution coefficients (*K*_*d*_) and separation factors $(\alpha^{a}_{b} )$ as a function of pH are calculated with the Eqs. (2) and (3) (Metwally et al. 2019):

2 $${K}_{d}\left(\mathrm{mL}/\mathrm{g}\right)=\left(\frac{{C}_{i}-{C}_{f}}{{C}_{f}}\right)\frac{V}{m}$$

3 $${\alpha }_{b}^{a}=\frac{{K}_{d}\left(a\right)}{{K}_{d}\left(b\right)}$$

where *C*_*i*_ and *C*_*f*_ are the initial and final concentrations of Cd(II)_,_ Co(II), Pb(II), Zn(II), and Sr(II) in solution, respectively. *V* is the volume of solution (mL) taken in (5 mL), and *m* is the mass of the sorbent (g) taken in (0.05), and *a* and *b* are two challenging types in a system.

### Saturation capacity

Repeated equilibration of 200 mg/L studied cations with the P(AA-AN)-talc in *V/m* = 200 mL/g was carried out for the saturation capacity determination at different heating temperatures (323–673 K). The mixtures were agitated in an agitator at 300 ± 1 K for 2 h. After equilibrium, the solution was separated, and the concentration of the metal ions was measured. This procedure was repeated many times with fresh solutions until the nanocomposite was saturated with metal ions. The saturation capacity *q* (mg/g) was calculated from Eq. (4) (Hassan et al. 2021; Ibrahim et al. 2021):

4 $$q=\left(\frac{C_i-C_e}m\right)X\;V$$

where *C*_*e*_ is the equilibrium concentration of Cd(II)_,_ Co(II), Pb(II), Zn(II), and Sr(II); *V* is the volume of solution (L) taken in (0.1 L); and *m* is the mass of P(AA-AN)-talc (g) taken in (0.05 g).

### Column separation

Chromatographic breakthrough investigations were directed as follows: 0.5 g of solid was packed in a plastic column (0.3 cm radius and 5 cm heights) to give a bed height of 1.1 cm^3^ volume. At a rated flow of 4 drops/min, 600 mL of 100 mg/L desired metal ions at pH = 4 was passed through the column beds. A breakthrough capacity (BTC) values were computed by the formula (Abass et al. 2021a):

5 $$\mathrm{Breakthrough}\;\mathrm{capacity}\left(\mathrm{mg}/\mathrm g\right)=\frac{V_{50\%}C_i}m$$

where *V*_50%_ is a volume for effluent at 50% breakthrough (L).

Double distilled water and different HNO_3_ concentrations (0.01, 0.05, 0.1, 0.2, and 0.5 M) were used to elute the loaded ions from the P(AA-AN)-talc surface at the flow rate of four drops/min, and the eluent was collected from every 20 min, and the concentrations were continuously measured.

## Results and discussion

### FTIR

**Fig. 1:**
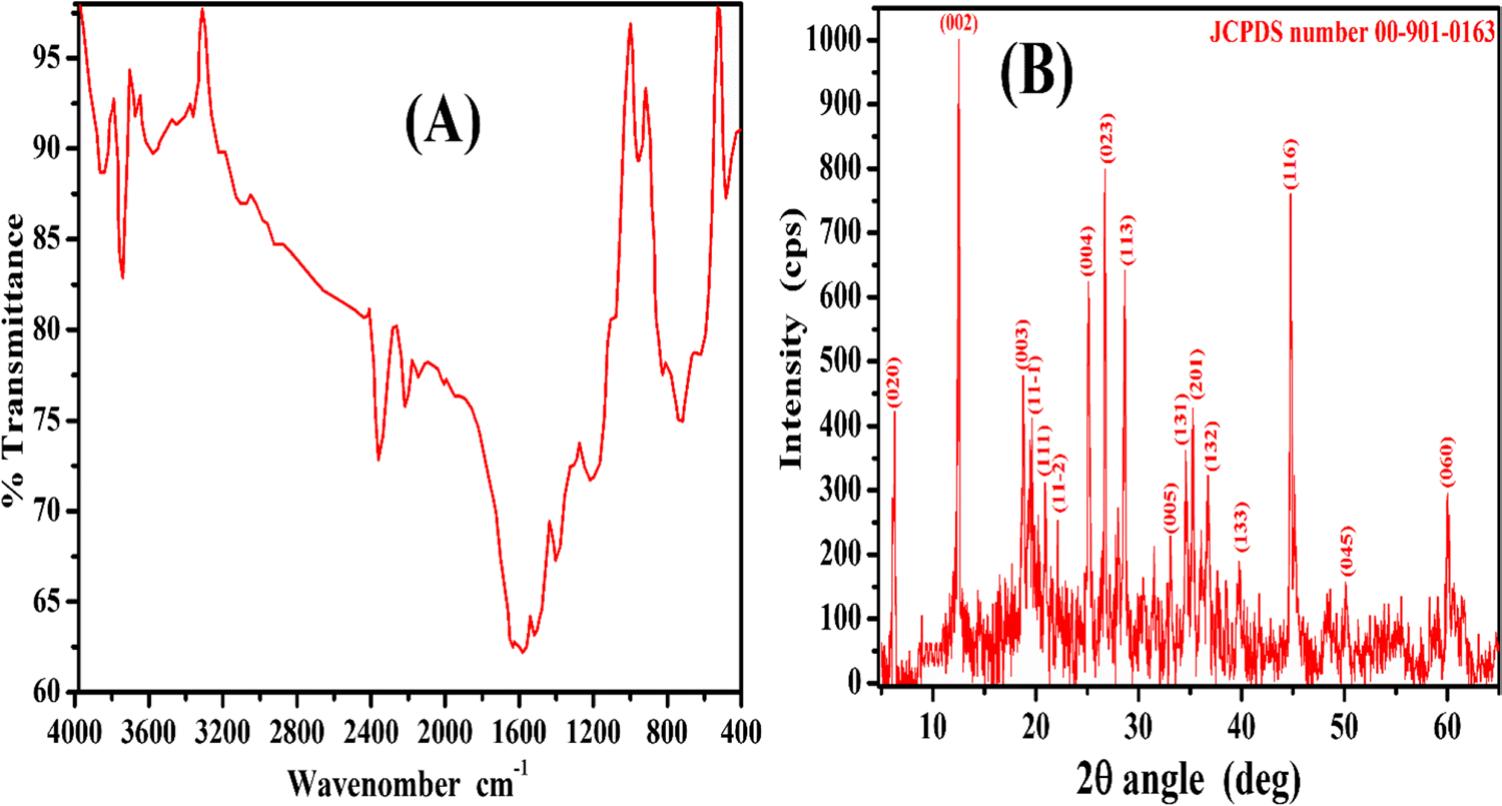
(**A**) IR spectrum and (**B**) XRD analysis for P(AA-AN)-talc nanocomposite

FTIR spectrum of P(AA-AN)-talc is shown in Fig. 1(A). This figure shows that bands at 3739 and 3568 cm^−1^ are due to H_2_O and OH absorbed on P(AA-AN)-talc (stretching vibration) (Abass et al. 2022b). A strong band at 2229 cm^−1^ is due to the C≡N bond of acrylonitrile (stretching vibration) (Lee et al. 2012a). Two bands at 1574 and 1511 cm^−1^ are due to N–O (stretching vibration) (Dawood and Li 2014). The band at 1395 cm^−1^ is due to O–H of carboxylic acid (bending vibration) (Lee et al. 2012b). The band at 1212 cm^−1^ is due to symmetric C-N (bending vibration) (Dawood and Li 2014). The band at 951 cm^−1^ is attributed to Si–O-H deformation vibration (Borai et al. 2015) or Si-CH_2_ (Mirzayev et al. 2021). The band at 722 cm^−1^ may be attributed to the Si–O-M (where M = Mg, Ca, and/or Al) (Nabi et al. 2011; Abass et al. 2022a). The band at 478 cm^−1^ may be attributed to the Si–H (Mirzayev et al. 2021). The three later bands deep-rooted the in-situ precipitation of talc (Mg, Al, and Si) in the net of a polymeric compound as seen later in EDX analysis.

### XRD analysis

XRD analysis of P(AA-AN)-talc nanocomposite is shown in Fig. 1(B). This figure indicated that P(AA-AN)-talc has a crystalline structure and exhibits many sharp peaks centered at 6.19˚, 18.64˚, 19.64˚, 20.9˚, 21.86˚, 24.94˚, 26.88˚, 28.36˚, 31.32˚, 34.61˚, 35.17˚, 36.7˚, 39.83˚, 44.67˚, 50.08˚, and 60.0˚ related to Miller index indications (020, 002, 003, 11–1, 111, 11–2, 004, 023, 113, 005, 131, 201, 132, 133, 116, 045, and 060), respectively, with JCPDS number 00–901-0163 confirming their crystalline nature with a monoclinic system, and this result was parallel to the XRD of chlorite (Zanazzi et al. 2007), Fe_3_O_4_/Talc nanocomposite (Thi Huong et al. 2020), and talcum powder (Wenlei et al. 2014).

### Thermal analysis

**Fig. 2:**
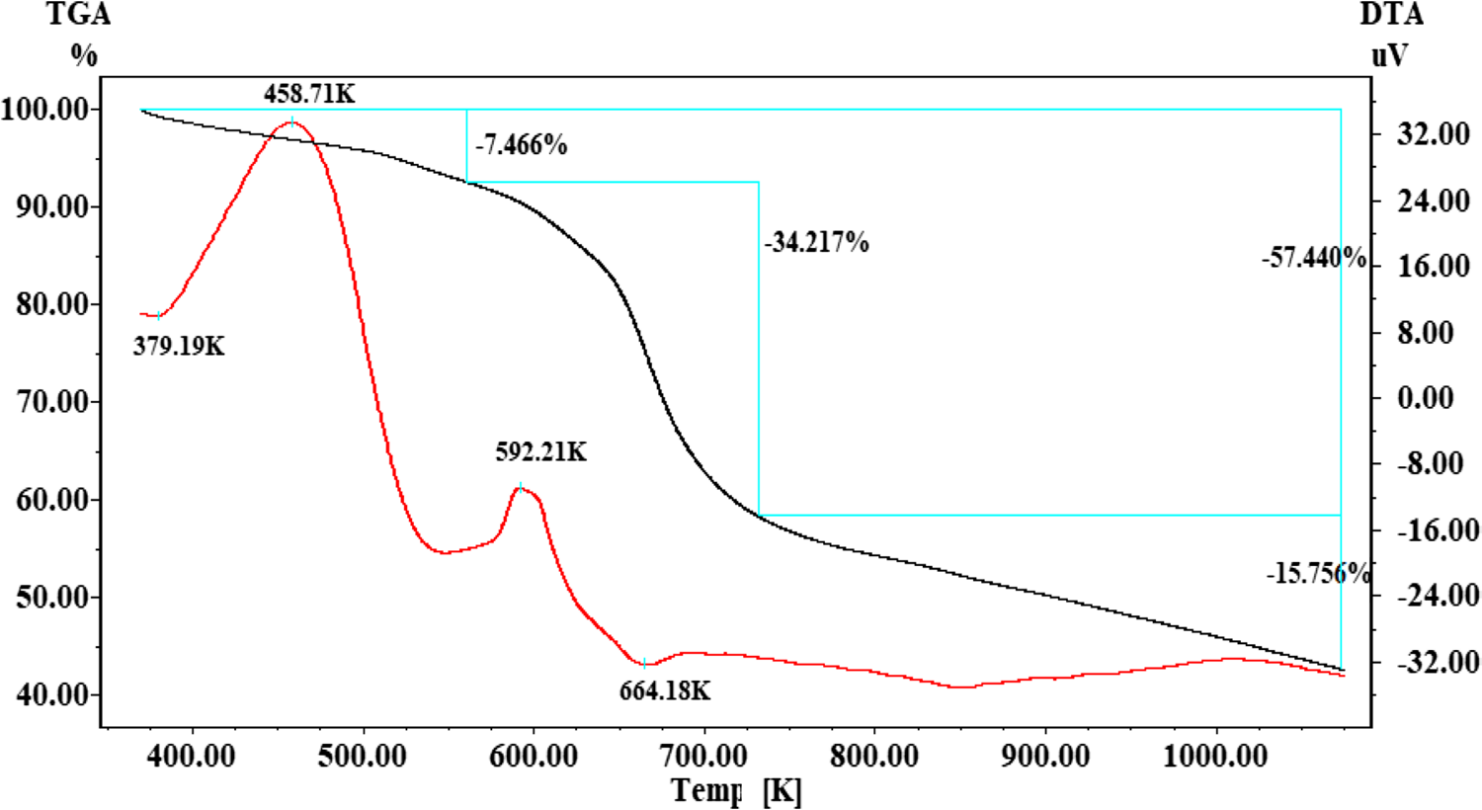
TGA and DTA analysis for P(AA-AN)-talc nanocomposite

The thermal analysis (TGA and DTA) of P(AA-AN)-talc nanocomposite (Fig. 2), showing that the weight loss process occurred via three steps. From 367 to 583 K due to the lessening of all moisture and free H_2_O of P(AA-AN)-talc (Hamoud et al. 2014), the lost weight is 7.5%. From 583 to 733 K due to subtraction of H_2_O of crystallization (Abass et al. 2021b) and complete decay of the organic part of P(AA-AN)-talc (El-Aryan et al. 2015), the lost weight is 34.2%. The third stage of weight loss (15.7%), which occurred from 733 to 1073 K caused by heating in N_2_ gas and the cycling reaction with C ≡ N bonds converted to C = N bonds (El-Aryan et al. 2015). DTA shows 2 peaks at 379 K and 664 K (endothermic), due to the subtraction of all surface and matrix-bound H_2_O from the polymeric compound and dehydration of carboxylic acid and decarboxylation of acrylic acid, respectively. Two exothermic peaks at 458 K and 592 K correspond to comprehensive decomposition of the organic molecule. From the data of TGA (Fig. 2), the losses weight is continued up to 1073 K. The full losses weight for P(AA-AN)-talc with the heating temperature is 57.7%.

#### SEM

**Fig. 3:**
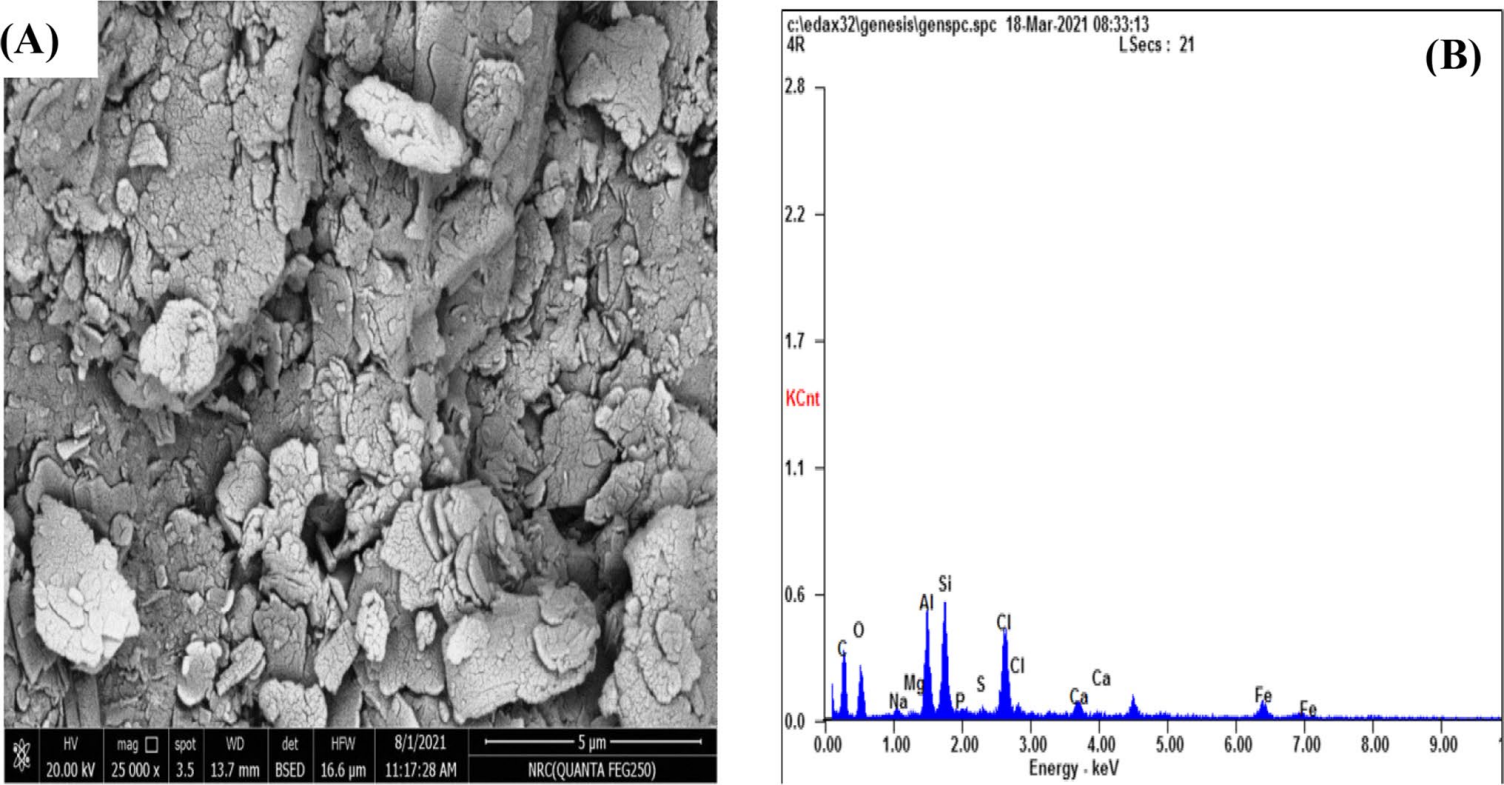
(**A**) SEM image and (**B**) EDX analysis of P(AA-AN)-talc nanocomposite

The performance of the particulate composite is detected by particle dispersion inside the matrix; a smooth particle distribution contributes to uniform powder properties, as it is shown in Fig. 3(A). Proper talc dispersion is noticed at diverse points of the sample cross sections with no presence of agglomeration. Also, a favored orientation of talc is appreciated for the composite, resulting from their plate-like structure (Andrić et al. 2014).

#### EDX

Figure 3(B) shows clearly distinct phases of Al, Mg, Si, O, and C, and the resulting relative percentages of different metals at the points are C = 55.3%, O = 20.2%, Si = 7.16%, Al = 6.3%, Mg = 0.29%, and Ca = 1.2. These results confirmed the impregnation of talc in polymeric resin layers as seen in the FTIR analysis mentioned earlier.

#### TEM

**Fig. 4:**
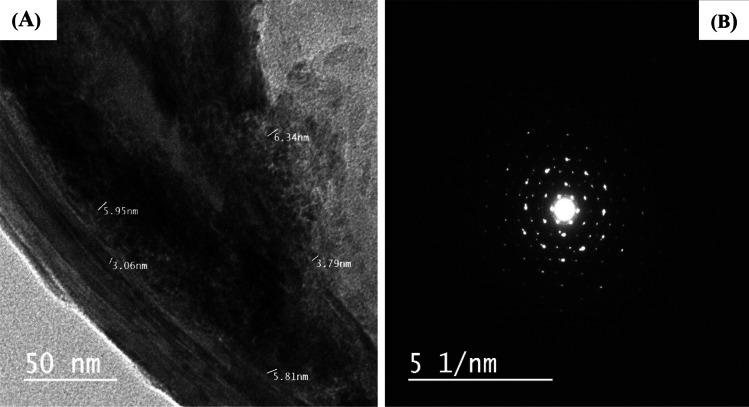
(**A**) TEM image and (**B**) SAED pattern for P(AA-AN)-talc nanocomposite

TEM images of P(AA-AN)-talc (Fig. 4(A)) were used to estimate small particle size and were extended from 3.1 to 6.34 nm. Also, P(AA-AN)-talc shows a spherical morphology. Also, the TEM image shows a good distribution of talc inside the polymeric resin. The selected area electron diffraction (SAED) pattern (Fig. 4(B)) shows the central, intense, and direct beam such as a pattern, with a sharply focused spot, confirming that P(AA-AN)-talc is at least partly crystalline (Carter et al. 1996). And these results confirm data calculated from XRD as mentioned earlier. The average particle size of the new material is 4.99 nm.

### Stability against chemicals

The chemical stability of P(AA-AN)-talc towards different solvents (H_2_O, HNO_3_, HCl, and NaOH) was performed and the results are tabulated in Table 2 and indicated that the prepared P(AA-AN)-talc is stable in H_2_O and HCl; also, the investigated sample is stable in HNO_3_ and NaOH up to 3 M; meanwhile, it starts to dissolve at 4 M and greatly dissolves at 6 M (Abou-Mesalam et al. 2018). Also, these results reveal that P(AA-AN)-talc is stable according to sequence order: HCl ˃ HNO_3_ ˃ NaOH.

**Table 2:** Chemical stability for P(AA-AN)-talc dissolved in different solvents for three days

The concentration of solvents, M	% Solubility			
	H_2_O	HCl	HNO_3_	NaOH
0.5	Below detection limit	1.92	4.83	6.6
1		10.7	11.9	15.4
2		12.8	16.2	19.85
3		17.3	21.0	22.6
4		22.0	27.2	34.9
6		47.3	PD	PD

*PD*, partially dissolved

### Point zero charge

**Fig. 5:**
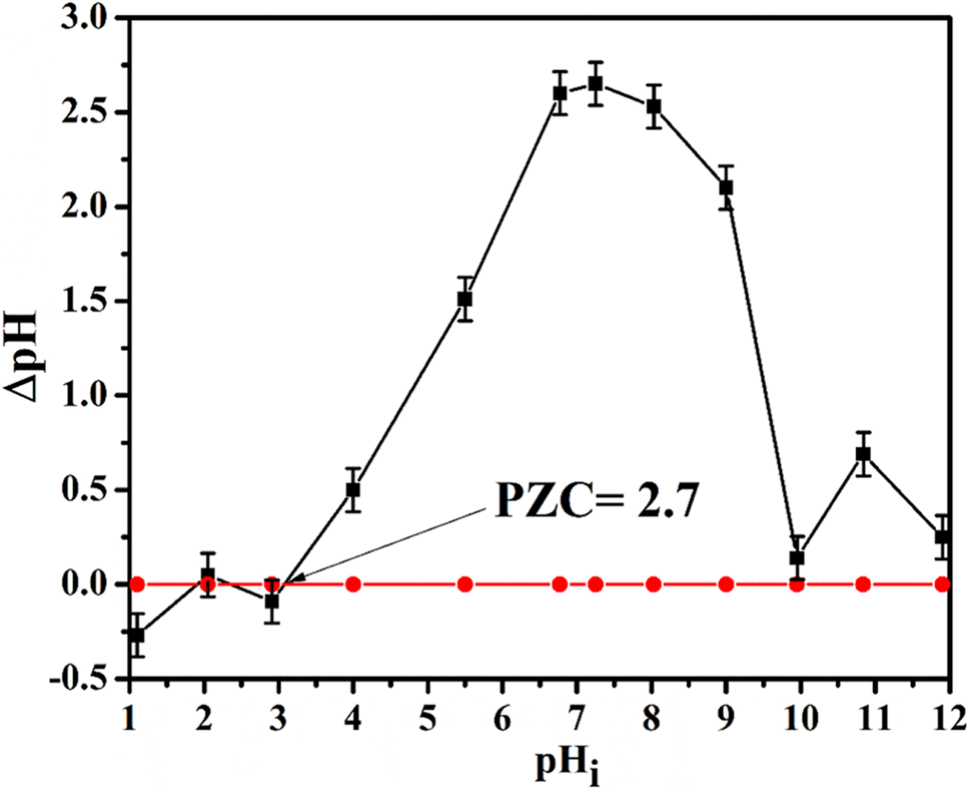
Plots of ΔpH vs pH_i_ for PZC

Point zero charge (PZC) for P(AA-AN)-talc was determined by using plots of ΔpH vs pHi (Fig. 5). From this Figure, it is clear that at acidic medium (pH < 7) the ∆pH increases with increasing pH_i_ then the ΔpH decreased at alkaline medium (pH > 7), and PZC was determined at pH value (2.7); this value revealed that the surface charge of P(AA-AN)-talc becomes neutral at this value, and above this value, it becomes negatively charged which leads to improve cationic species, whereas the surface of P(AA-AN)-talc is positively charged below this pH value which leads to improve anionic species.

## Adsorption investigations

### Distribution coefficients (K_d_)

**Fig. 6:**
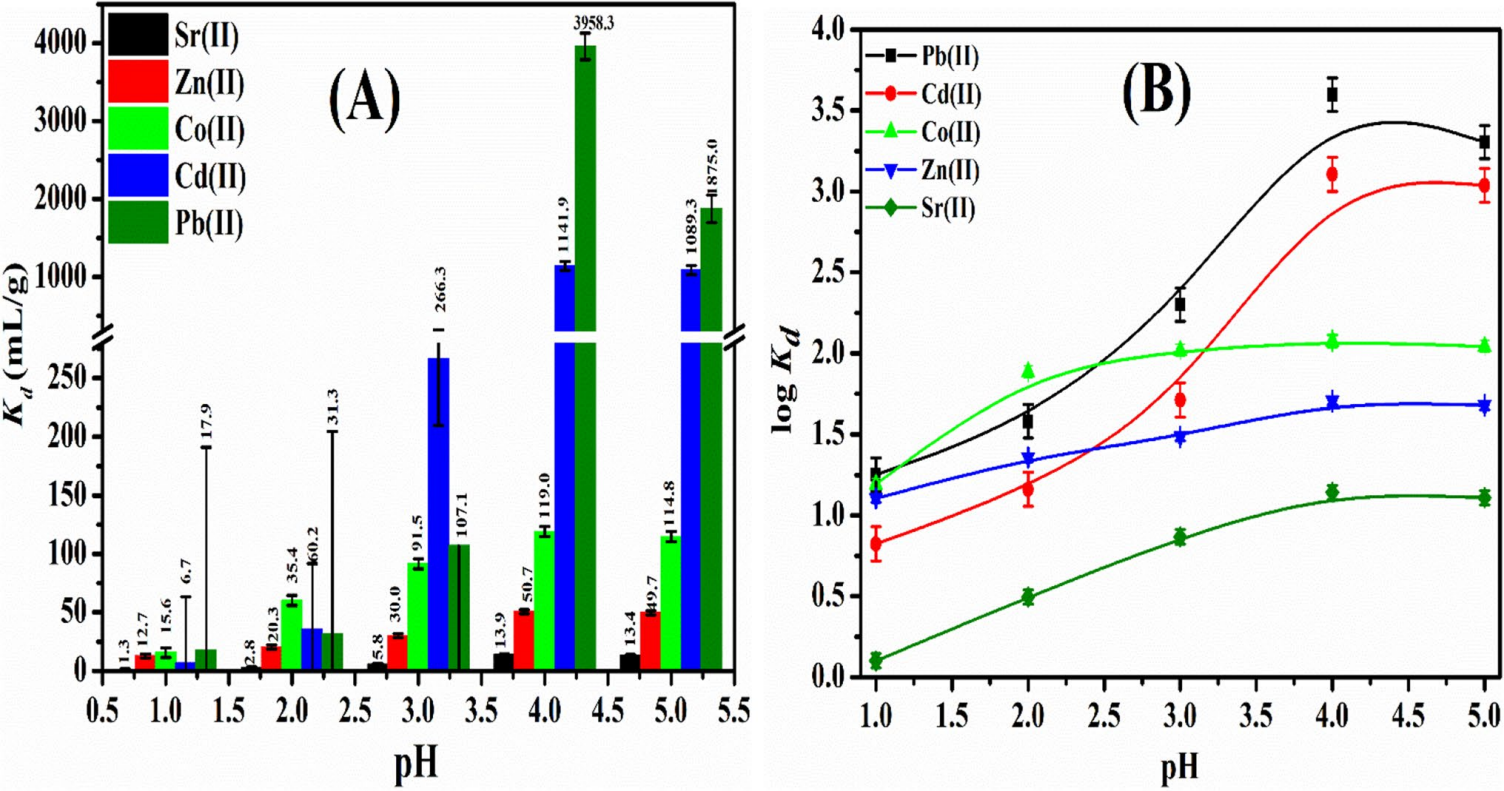
Sorption behavior of Pb(II), Cd(II), Co(II), Zn(II), and Sr(II) sorbed onto P(AA-AN)-talc at 300 ± 1 K. (**A**) Effect of pH on *K*_*d*_ and (**B**) plots of *log K*_*d*_ against pH

Figure 6(A) shows the *K*_*d*_ difference of Pb^2+^, Cd^2+^, Co^2+^, Zn^2+^, and Sr^2+^ (100 mg/L) on P(AA-AN)-talc in H^+^ form, as a function of pH. The *K*_*d*_ improves with the increase in pH. At lower pH lower than three, *K*_*d*_ of the studied cations was inhibited; this is attributed to the existence of excess protons competing with Pb^2+^, Cd^2+^, Co^2+^, Zn^2+^, and Sr^2+^ in the solution and preferably occupying the binding sites existing in P(AA-AN)-talc (Metwally et al. 2019). At pH ≥ 3, *K*_*d*_ continuously increases with the increase in pH due to the decline in proton competition and optimum uptake achieved at pH 4 as well as uptake was slightly decreased at pH higher than 4, due to the studied cations starting to precipitate, and all experimental work was done at pH 4. Distribution coefficients (*K*_*d*_) and separation factors $(\alpha^{a}_{b} )$ for the mentioned cations in different pHs (1–5) are computed and presented in Table 3. The results reveal that *K*_*d*_ values have the sequence: Pb^2+^  > Cd^2+^  > Co^2+^  > Zn^2+^  > Sr^2+^ reflecting that the sorption of metal ions was achieved in hydrated ionic radii except Sr^2+^ applied in unhydrated ionic radii (Pb^2+^, Cd^2+^, Co^2+^, Zn^2+^, and Sr^2+^ have ionic radii 1.2, 0.97, 0.63, 0.74, and 1.13 Å, respectively) according to this sequence (Abou-Mesalam et al. 2020). Separation factor $(\alpha^{a}_{b} )$ values were computed and revealed that Pb^2+^ has very higher $\alpha^{a}_{b}$ by 284.8, 78.1, 33.3, and 3.5 for Sr^2+^, Zn^2+^, Co^2+^, and Cd^2+^ at optimum uptake (pH 4); these values indicated that Pb(II) ion can very easily be separated from radioactive and industrial waste solutions, which reflected no selectivity for Sr^2+^. Non-linear relations between *log K*_*d*_ and pH were observed for studied cations as exposed in Fig. 6(B). The non-ideality of the exchange reaction was clarified from this relation. The difference may be due to the eminence of a mechanism other than ion exchange, such as precipitation and surface sorption (Abou-Mesalam et al. 2018).

**Table 3:** Distribution coefficients *(K*_*d*_*)* and separation factors $(\alpha^{a}_{b} )$ for Pb(II), Cd(II)_,_ Co(II), Zn(II), and Sr(II) sorbed onto P(AA-AN)-talc at 300 ± 1 K

pH	*K*_*d*_ (mL/g) and $\alpha^{a}_{b}$	Sr(II)	Zn(II)	Co(II)	Cd(II)	Pb(II)
1	*K*_*d*_	1.3	12.7	15.6	6.7	17.9
	$\alpha^{a}_{b}$		9.8	12.0	5.2	13.8
				1.2	0.5	1.4
					0.4	1.1
						2.7
2	*K*_*d*_	2.8	20.3	60.2	35.4	31.3
	$\alpha^{a}_{b}$		7.3	21.5	12.6	11.2
				3.0	1.7	1.5
					0.6	0.5
						0.9
3	*K*_*d*_	5.8	30.0	91.5	266.3	107.1
	$\alpha^{a}_{b}$		5.2	15.8	45.9	18.5
				3.1	8.9	3.6
					2.9	1.2
						0.4
4	*K*_*d*_	13.9	50.7	119.0	1141.9	3958.3
	$\alpha^{a}_{b}$		3.6	8.6	82.2	284.8
				2.3	22.5	78.1
					9.6	33.3
						3.5
5	*K*_*d*_	13.4	49.7	114.8	1089.3	1875.0
	$\alpha^{a}_{b}$		3.7	8.6	81.3	139.9
				2.3	21.9	37.7
					9.5	16.3
						1.7

### Agitating time impact

**Fig. 7:**
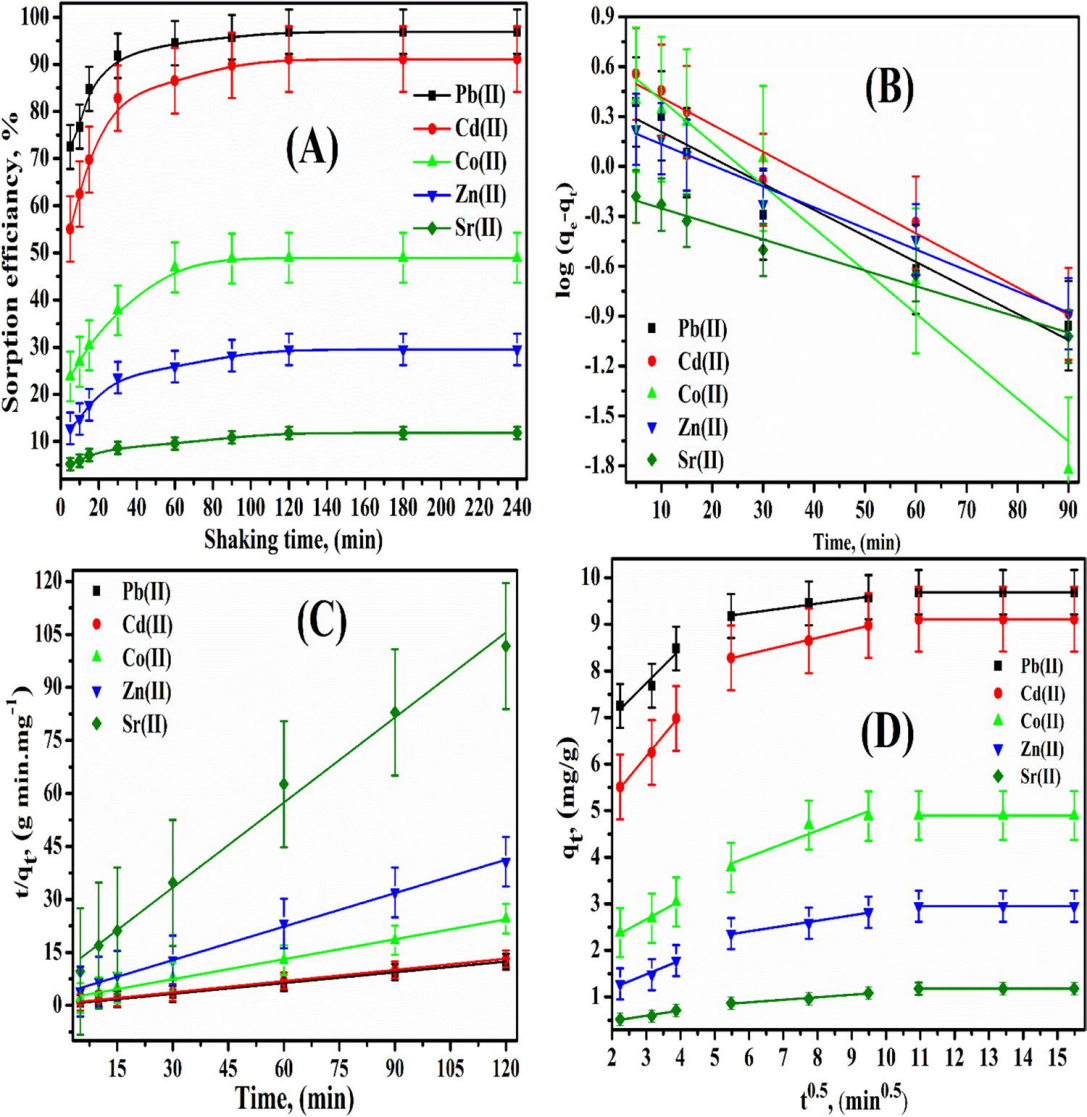
Sorption behavior of Pb(II), Cd(II), Co(II), Zn(II), and Sr(II) sorbed onto P(AA-AN)-talc at 300 ± 1 K. (**A**) Effect of shaking time, (**B**) pseudo-first-order kinetic, (**C**) pseudo-second-order kinetic, and (**D**) plot of *q*_*t*_ vs. *t*^*0.5*^

The influence of shaking time changed from 5 to 240 min on the sorption efficiency of Pb^2+^, Cd^2+^, Co^2+^, Zn^2+^, and Sr^2+^ (100 mg/L) onto P(AA-AN)-talc was done at pH 4, and the experimental results are given in Fig. 7(A), as a relation between sorption efficiency percent and time. From this figure, it is clear that, as the mixing time increases from 5 to 120 min, the sorption percent of Pb^2+^, Cd^2+^, Co^2+^, Zn^2+^, and Sr^2+^ increased from about 72.5 to 96.9% for Pb^2+^, from about 55.1 to 91.1% for Cd^2+^, from about 23.8 to 48.95% for Co^2+^, from about 12.8 to 29.5% for Zn^2+^, and from about 5.2 to 11.8% for Sr ^2+^. Further, an increase in the mixing time up to 240 min does not affect the removal of Pb^2+^, Cd^2+^, Co^2+^, Zn^2+^, and Sr^2+^. This means that the sorption equilibrium has been achieved at 2 h. Therefore, 2 h represents the preferred time to maximize the Pb^2+^, Cd^2+^, Co^2+^, Zn^2+^, and Sr^2+^ sorption using the P(AA-AN)-talc, and this time was used for all experiments.

### Saturation capacity and thermal stability

The influence of heating temperature on the capacity of studied cations on P(AA-AN)-talc in H^+^ form is detected at 300 ± 1 K, and the data are presented in Table 4. The prominent reduction in the capacity was observed with rising temperature; it is related to the decay of the organic part of the polymeric material with an increase in temperatures as exposed in TGA and DTA data (Gupta et al. 2015). The capacities of studied cations have sequence order: Pb^2+^  > Cd^2+^  > Co^2+^  > Zn^2+^  > Sr^2+^; this order reveals that the sorption process was achieved according to the lessening in the hydrated ionic radii and hydration energy (Gupta et al. 2003; Rahman et al. 2017). The high capacity of the material investigated for Pb^2+^ and Cd^2+^ may be due to the higher complexing ability of these ions (Abou-Mesalam et al. 2020).

**Table 4:** Effect of temperature on the saturation capacity of P(AA-AN)-talc for various metal ions

Heating temperature, K	Weight loss (%)	Pb(II)		Cd(II)		Co(II)		Zn(II)		Sr(II)	
		S.C.	%R	S.C.	%R	S.C.	%R	S.C.	%R	S.C.	%R
323	Nil	55.9	100	40.1	100	32.6	100	26.1	100	14.3	100
373	5.5	48.7	87.1	36.1	90.2	31.0	95.1	24.4	93.5	13.7	95.8
473	6.6	48.4	86.6	33.8	84.3	27.4	84.0	16.5	63.2	12.4	86.7
673	45.7	43.0	76.9	31.4	78.3	26.5	81.3	15.4	59.0	11.5	80.4

***S.C*.** is the saturation capacity (mg/g) and % *R* is the retention of saturation capacity

### Kinetic investigation

The pseudo-first-order and pseudo-second-order equations are specified as (Gürses et al. 2014):

6 $$log\left({q}_{e}-{q}_{t}\right)=-\left(\frac{{K}_{f}}{2.303}\right)t+log {q}_{e}$$

7 $$\frac{t}{{q}_{t}}=\frac{1}{{K}_{s}{q}_{e}^{2}}+\frac{t}{{q}_{e}}$$

where *K*_*f*_ (min^−1^) and *K*_*s*_ (g mg^−1^ min^−1^) are the rates constant of pseudo-first-order and pseudo-second-order, respectively, and *q*_*e*_ and *q*_*t*_ (mg/g) are the amounts sorbed per gram at equilibrium and time *t*. Plotting *log (q*_*e*_*-q*_*t*_*)* and *t* as shown in Fig. 7(B), the plot shows a linear relationship. From Table 5, it is found that the pseudo-first-order model does not apply to Pb^2+^, Cd^2+^, Co^2+^, Zn^2+^, and Sr^2+^ sorption onto P(AA-AN)-talc. When the *q*_*e*_ calculated from pseudo-first-order plots was compared with the *q*_*e*_ (experimental) considered, one of the main discrepancies was observed (Sheha and El-Zahhar 2008). However, pseudo-second-order is obtained from plotting *t/q*_*t*_ against *t* for Pb^2+^, Cd^2+^, Co^2+^, Zn^2+^, and Sr^2+^ sorption as shown in Fig. 7(C), and it is found that the relationship is linear. The data represented in Table 5 reveal that the correction coefficients (*R*^*2*^) are closed to unity which indicates that the sorption procedure follows the pseudo-second-order model; also, the values (*q*_*e*_, *K*_*f*_, and *K*_*s*_) confirm that the pseudo-second-order model applies to Pb^2+^, Cd^2+^, Co^2+^, Zn^2+^, and Sr^2+^ sorption onto P(AA-AN)-talc. Similar phenomena have been observed for Pb(II) and Cd(II) adsorption on biochars (Park et al. 2013), Sr(II), and Cs(I) sorbed by irradiated saccharomyces cerevisiae (Tan et al. 2017), cobalt(II) sorbed by orange peel waste (Altunkaynak et al. 2021), and adsorption of Zn(II) on natural bentonite (Sen and Gomez 2011).

**Table 5:** Kinetic parameters of various metal ions onto P(AA-AN)-talc at 300 ± 1 K

Metal ions sorbed	Pseudo-1st-order			Pseudo-2nd-order			*q*_*e*,_ exp. (mg/g)
	*K*_*f*_ × 10^−3^	*q*_*e*_, (mg/g)	*R*.^*2*^	*K*_*S*_ × 10^−3^	*q*_*e*_, (mg/g)	*R*^*2*^	
Pb(II)	15.7	2.32	0.94	9.7	10.309	0.999	9.69
Cd(II)	16.3	3.78	0.968	10.6	9.438	0.999	9.11
Co(II)	25.7	4.53	0.963	18.8	5.313	0.997	4.9
Zn(II)	12.7	1.82	0.976	31.6	3.166	0.997	2.95
Sr(II)	9.4	0.69	0.973	80.2	1.246	0.991	1.18

### Mechanism of adsorption

The adsorption mechanism was examined using of intra-particle diffusion model (Karaca et al. 2013; Dakroury et al. 2021).

8 $${q}_{t}={K}_{id}{t}^{0.5}+C$$

where *C* (intercept) and *K*_*id*_ (slope) is the intra-particle diffusion rate constant (mg min^−0.5^ g^−1^). The intra-particle diffusion model for the sorption process of the studied metal ions was established through three steps (Karaca et al. 2013; Dakroury et al. 2021). The first stage includes the diffusion of metal ions from the solution to the surface of the P(AA-AN)-talc (from 5 to 15 min). The second stage (from 30 to 90 min) describes the gradual sorption on the surface of the P(AA-AN)-talc, which may be the rate-limiting step. The third stage (from 120 to 240 min) is the equilibrium saturation. The relation between *q*_*t*_ and *t*^*0.5*^ is represented in Fig. 7(D), and the three steps of the sorption mechanism are observed in this figure. The intercept which is the thickness of the surface gave information about the contribution of the surface sorption in the rate-determining step. The larger the intercept, the greater its contribution. Table 6 shows the parameters obtained from the second part of the linear plot. The sorption mechanism of Pb^2+^, Cd^2+^, Co^2+^, Zn^2+^, and Sr^2+^ onto P(AA-AN)-talc was found to be rapid at the initial period of contact time and then to become constant with the increase in contact time. The multi-diffusion step is the main factor in the control of the sorption mechanism, which includes both film and intra-particle diffusion.

**Table 6:** Diffusion models parameters for sorption of various metal ions onto P(AA-AN)-talc at 300 ± 1 K

Metal ions sorbed	Time range, min	*K*_*id*_, g mg^−1^ min.^−1^	*C*	*R*.^*2*^
Pb(II)	5–15	0.73723	5.52495	0.87966
	30–90	0.10071	8.64095	0.96964
	120–240	-	9.69	-
Cd(II)	5–15	0.89314	3.48647	0.98967
	30–90	0.17401	7.31939	0.99632
	120–240	-	9.11	-
Co(II)	5–15	0.39982	1.46773	0.97553
	30–90	0.2806	2.32585	0.84142
	120–240	-	4.895	-
Zn(II)	5–15	0.30103	0.58301	0.92805
	30–90	0.11454	1.7216	0.98774
	120–240	-	0.55917	-
Sr(II)	5–15	0.11407	0.25413	0.89901
	30–90	0.11407	0.25413	0.89901
	120–240	-	0.55917	-

### Sorption isotherms

**Fig. 8:**
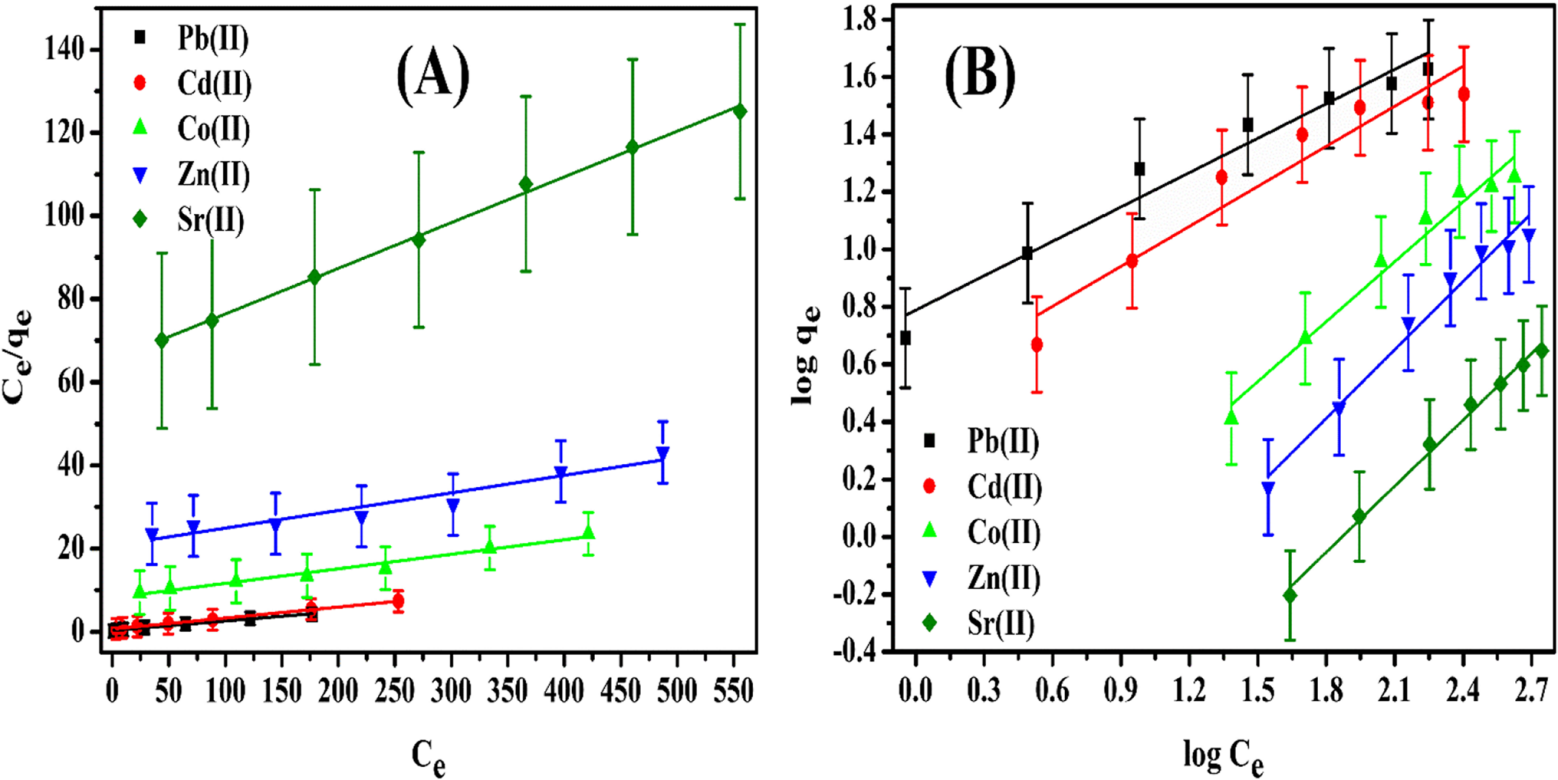
Sorption behavior of Pb(II), Cd(II), Co(II), Zn(II), and Sr(II) sorbed onto P(AA-AN)-talc at 300 ± 1 K. (**A**) Langmuir isotherm and (**B**) Freundlich isotherm

The Langmuir equation relates the concentration of a medium above a solid phase surface at a constant temperature to cover the sorbed molecules on the solid phase surface (Hamed et al. 2016a). The Langmuir isotherm model suggests an estimation of the maximum adsorption capacity that occurred by complete monolayer adsorption on the adsorbent surface (Hamed 2014). The Langmuir isotherm can be represented in the next equation (Hamed et al. 2016a):

9 $$\frac{{C}_{e}}{{q}_{e}}=\frac{{C}_{e}}{{Q}_{max}}+\frac{1}{{bQ}_{max}}$$

where *C*_*e*_ is the equilibrium concentration (mg/L), *Q*_*ma*x_ is monolayer capacity (mg/g), and *b* is the sorption equilibrium constant related to the energy of sorption. The represented relation between *C*_*e*_*/q*_*e*_ and *C*_*e*_ gives straight lines for both Pb^2+^, Cd^2+^, Co^2+^, Zn^2+^, and Sr^2+^ sorbed onto P(AA-AN)-talc nanocomposite as shown in Fig. 8. Table 7 represents the data obtained from the linear form of the Langmuir equation. The correlation coefficients (*R*^*2*^) are 0.992, 0.998, 0.976, 0.991, and 0.996 for Pb^2+^, Cd^2+^, Co^2+^, Zn^2+^, and Sr^2+^, respectively. The maximum monolayer capacities (*Q*_*max*_) obtained from the Langmuir model were (43.8, 37.91, 28.6, 23.6, and 9.09) for Pb^2+^, Cd^2+^, Co^2+^, Zn^2+^, and Sr^2+^, respectively.

**Table 7:** Isotherm parameters for sorption of Pb(II), Cd(II)_,_ Co(II), Zn(II), and Sr(II) sorbed onto P(AA-AN)-talc at 300 ± 1 K

Metal ions sorbed	Langmuir constants				Freundlich constants		
	*Q*_*max*_ (mg/g)	*b* (L/mg)	*R*_*L*_	*R*.^*2*^	*1/n*	*K*_*F*_ (mg/g)	*R*.^*2*^
Pb(II)	43.80	0.075	0.04	0.992	0.40	6.14	0.957
Cd(II)	37.91	0.040	0.06	0.998	0.46	3.33	0.915
Co(II)	28.60	0.004	0.39	0.976	0.70	0.31	0.970
Zn(II)	23.60	0.002	0.57	0.991	0.79	0.10	0.975
Sr(II)	9.09	0.002	0.62	0.997	0.77	0.04	0.993

The separation factor (*R*_*L*_) considers one of the essential characteristics of the Langmuir isotherm model (Hamed et al. 2016a), which can be calculated from the Langmuir constant, *b*, as the following:

10 $${R}_{L}=\frac{1}{1+{bC}_{o}}$$

From data present in Table 7, *R*_*L*_ values were found to be 0 < *R*_*L*_ < 1 reflecting the favorable sorption isotherms of Pb^2+^, Cd^2+^, Co^2+^, Zn^2+^, and Sr^2+^ (Hamed et al. 2016a).

The Freundlich isotherm model is an empirical formula that accurately represents multi-layer sorption on heterogeneous surfaces (Abass et al. 2021a). A Freundlich equation can be represented in linear form as:

11 $$\mathrm{log}{q}_{e}=\mathrm{log}{K}_{F}+\frac{1}{n}\mathrm{log}{C}_{e}$$

where *K*_*F*_ (mg/g) and *1/n* are Freundlich constants related to adsorption capacity and adsorption intensity. These constants are calculated from both slope and intercept of the linear plot of *log q*_*e*_ versus *log C*_*e*_, respectively. The sorption results of Pb^2+^, Cd^2+^, Co^2+^, Zn^2+^, and Sr^2+^ onto P(AA-AN)-talc nanocomposite performed by Freundlich isotherms are shown in Fig. 8. Freundlich isotherm parameters (*K*_*F*_, *1/n*, and *R*^*2*^) are represented in Table 7. The values of *R*^*2*^ = 0.959, 0.915, 0.97, 0.974, and 0.993 for Pb^2+^, Cd^2+^, Co^2+^, Zn^2+^, and Sr^2+^, respectively, were much lower than the Langmuir isotherm values, reflecting the applicability of Langmuir than Freundlich isotherm.

### Comparison of monolayer capacity with different sorbents reported in the literature

The monolayer capacities (*Q*_*max*_) of P(AA-AN)-talc for the sorption of Pb^2+^, Cd^2+^, Co^2+^, Zn^2+^, and Sr^2+^ was compared with other sorbents reported in the literature. As represented in Table 8, the monolayer capacities of P(AA-AN)-talc toward the studied cations are higher than the previously reported values which suggested that P(AA-AN)-talc is a promising sorbent to remove Pb^2+^, Cd^2+^, Co^2+^, Zn^2+^, and Sr^2+^ from aqueous solutions (Annadurai et al. 2003; Anitha et al. 2015; Ghasemi et al. 2018; Abou-Lilah et al. 2020; Hassan et al. 2020; Herrera et al. 2020; Abass et al. 2021a, 2022c; Kayranli 2021; Alsehli 2021; Abou-Mesalam et al. 2022).

**Table 8:** Comparison of the monolayer capacity of Pb(II), Cd(II)_,_ Co(II), Zn(II), and Sr(II) sorbed onto various sorbents

Sorbents	Capacity (mg/g)					Ref. no
	Pb(II)	Cd(II)	Co(II)	Zn(II)	Sr(II)	
P(AA-AN)-talc	43.8	37.91	28.6	23.6	9.09	Current work
Orange and banana peels	7.97	NR	2.55	34.71	NR	(Annadurai et al. 2003)
Leaf samples of Indian almond	27.5	42.5	NR	NR	NR	(Alsehli 2021)
PB-Al_2_O_3_ nanoparticles	NR	18.40	NR	NR	NR	(Herrera et al. 2020)
LP-Al_2_O_3_ nanoparticles	NR	18.66	NR	NR	NR	(Herrera et al. 2020)
CC-Al_2_O_3_ nanoparticles	NR	18.20	NR	NR	NR	(Herrera et al. 2020)
CoTiSi	NR	10.96	16.02	NR	NR	(Abou-Mesalam et al. 2022)
Raw bentonite	NR	NR	25.0	NR	14.29	(Abou-Lilah et al. 2020)
DOP-TETA-MNP	NR	NR	NR	6.49	NR	(Ghasemi et al. 2018)
Chitosan–PVA Blend	NR	NR	NR	5.91	NR	(Anitha et al. 2015)
Pistachio	NR	NR	NR	59.52	NR	(Kayranli 2021)
Peanut	NR	NR	NR	54.64	NR	(Kayranli 2021)
Almond	NR	NR	NR	51.81	NR	(Kayranli 2021)
*Salvadora persica*	NR	NR	NR	NR	41.49	(Hassan et al. 2020)
ZrSnP	NR	NR	NR	NR	17.7	(Abass et al. 2022c)
PAN/BC nanocomposite	NR	NR	15.8	NR	17.7	(Abass et al. 2021a)

## Thermodynamic studies

**Fig. 9:**
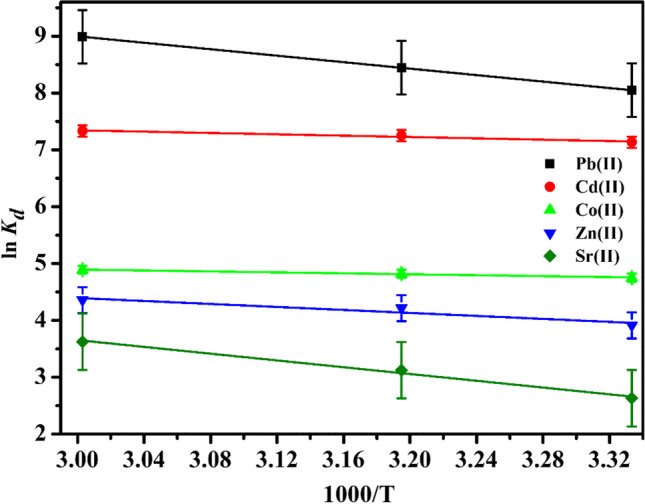
Van’t Hoff plot of the adsorption of Pb(II), Cd(II)_,_ Co(II), Zn(II), and Sr(II) on P(AA-AN)-talc

A linear relation between *ln K*_*d*_ of studied cations on P(AA-AN)-talc and *1000/T* along with the Van’t Hoff relation (Abdel-Galil et al. 2016) is exposed in Fig. 9:

12 $$\mathrm{ln}{K}_{d}=\frac{{\Delta S}^{^\circ }}{R}-\frac{{\Delta H}^{^\circ }}{RT}$$

where (*∆S˚*, *∆H˚*, *R*, and *T*) are the entropy change, enthalpy change, the universal gas constant, and absolute temperature. The *K*_*d*_ of the investigated cations improved with increasing temperature from 300 to 333 K (i.e., the *K*_*d*_ reduced with rising *1000/T*) and similar phenomena for adsorption of Remazol Red RB by modified clay (Karaca et al. 2013). This enhancement in adsorption with increasing the temperature is corresponding to the acceleration of some originally slow adsorption phases and the creation of some fresh exchangeable sites on the adsorbent layers (Abdel-Galil et al. 2016). From both slopes and intercepts of linear relation displayed in Fig. 9, *∆H˚* and *∆S˚* are computed and tabulated in Table 9. The positive values of both *∆H˚* and *∆S˚* indicate the endothermic nature and increased randomness of solid solution interface during the adsorption of these cations on P(AA-AN)-talc, respectively (El-Naggar et al. 2010; Abdel-Galil et al. 2016).

**Table 9:** Thermodynamic parameters for adsorption of Pb(II), Cd(II)_,_ Co(II), Zn(II), and Sr(II) on P(AA-AN)-talc

Metals	Temp., K	*∆H˚*(kJ/mol)	*∆S˚*(J/mol.K)	*∆G˚*(kJ/mol)
Pb(II)	300	23.6	145.7	− 20.1
	313			− 21.9
	333			− 24.9
Cd(II)	300	4.9	75.7	− 17.8
	313			− 18.8
	333			− 20.3
Co(II)	300	3.5	51.1	− 11.9
	313			− 12.5
	333			− 13.6
Zn(II)	300	10.9	69.3	− 9.9
	313			− 10.8
	333			− 12.2
Sr(II)	300	24.8	104.8	− 6.6
	313			− 8.0
	333			− 10.1

The free energy change of adsorption *∆G˚* was computed by the relation:

13 $$\Delta G^\circ =\Delta H^\circ -T\Delta S^\circ$$

The negative values of *∆G˚* represented in Table 9 reflect that the sorption process is spontaneous and indicates the better sorption of these ions on P(AA-AN)-talc compared with H^+^ ion (El-Naggar et al. 2010).

## Column investigations

### Binary system

**Fig. 10:**
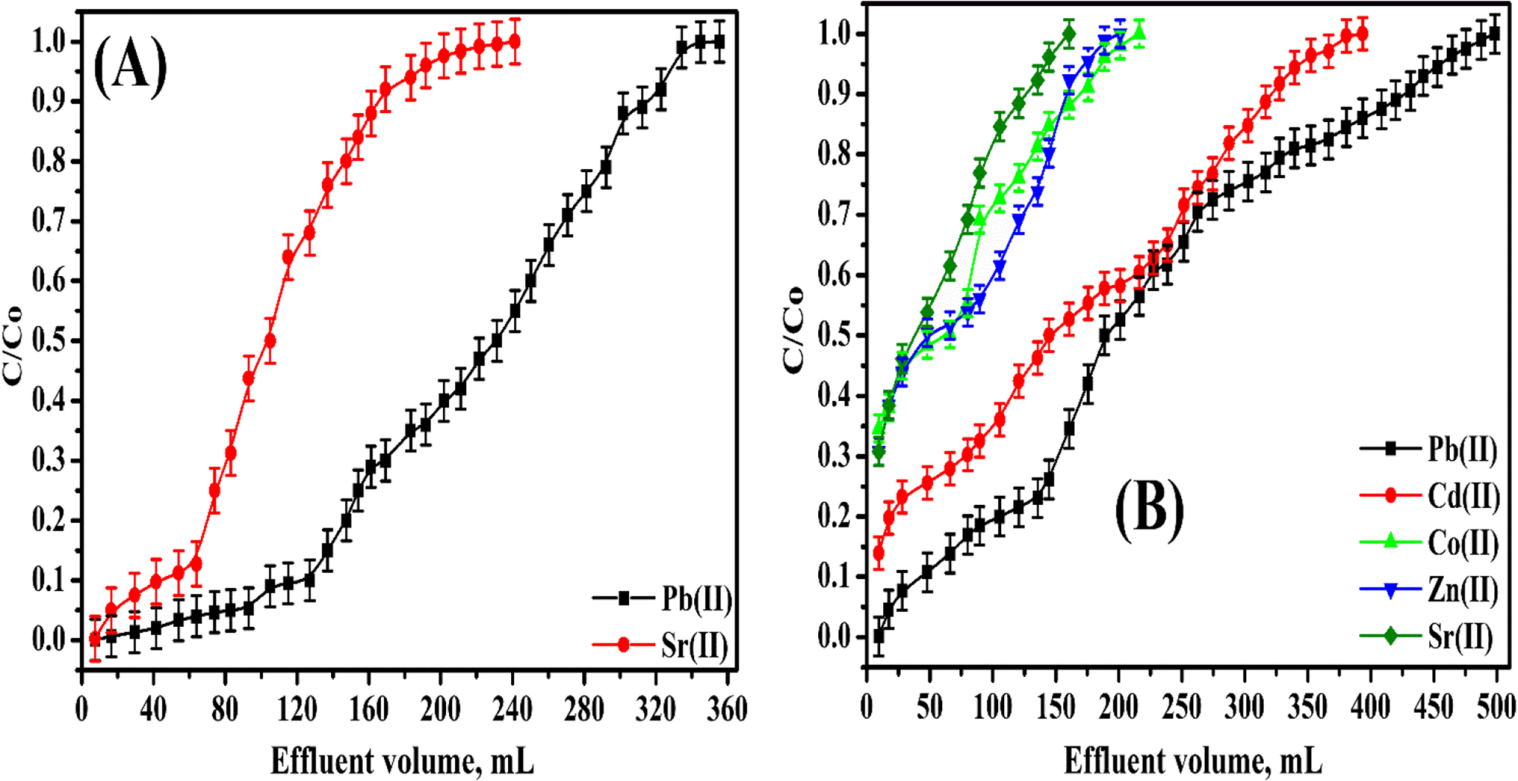
Breakthrough capacities of (**A**) binary system Pb(II) and Sr(II) and (**B**) multi-component system Pb(II), Cd(II), Co(II), Zn(II), and Sr(II) sorbed onto P(AA-AN)-talc at pH 4

Figure 10(A) shows breakthrough curves for Pb^2+^ and Sr^2+^ (100 mg/L) onto the P(AA-AN)-talc column. The concentrations of respective ions in the effluent to the feed solution (*C/C*_*o*_) vs. effluent volume, *V* (L) are plotted on a breakthrough curve. The uptake of Pb^2+^ and Sr^2+^ per gram for solid is computed from Fig. 10(A) using Eq. (5) and found to be 46.3 and 25.0 mg/g for Pb^2+^ and Sr^2+^, respectively. From these consequences, it is found that selectivity followed order: Pb(II) >  > Sr(II). This order proved that the sorption process was taking place for Pb^2+^ and Sr^2+^ in hydrated ionic radii, similar to the result in the batch method (Abou-Mesalam et al. 2018). And a BTC is lower than the capacity determined from a batch method, due to the fast dynamic motion of investigated cations. From the data above-mentioned, the conclusions can be expected that P(AA-AN)-talc is suitable for the capture of Pb^2+^ from Sr^2+^ at pH = 4.

### Multi-component system

The breakthrough curves for Pb^2+^, Cd^2+^, Co^2+^, Zn^2+^, and Sr^2+^ (100 mg/L for each) onto the P(AA-AN)-talc column are displayed in Fig. 10(B). A breakthrough capacity is computed as mentioned earlier in the binary system from Fig. 10(B) using Eq. (5) and found to be 37.7, 28.9, 13.2, 9.6, and 7.4 mg/g for Pb^2+^, Cd^2+^, Co^2+^, Zn^2+^, and Sr^2+^, respectively. From these data, the selectivity followed order: Pb^2+^  > Cd^2+^  > Co^2+^  > Zn^2+^  > Sr^2+^. This order proved that the sorption was achieved in hydrated ionic radii; hence, the breakthrough capacity was lower than that obtained by the batch technique, due to the fast dynamic motion of studied cations. From the data above-mentioned, the conclusion can be supposed that P(AA-AN)-talc is a more suitable sorbent for the uptake of all studied cations from liquid solutions in the pH = 4.

## Elution

### Elution of binary system

**Fig. 11:**
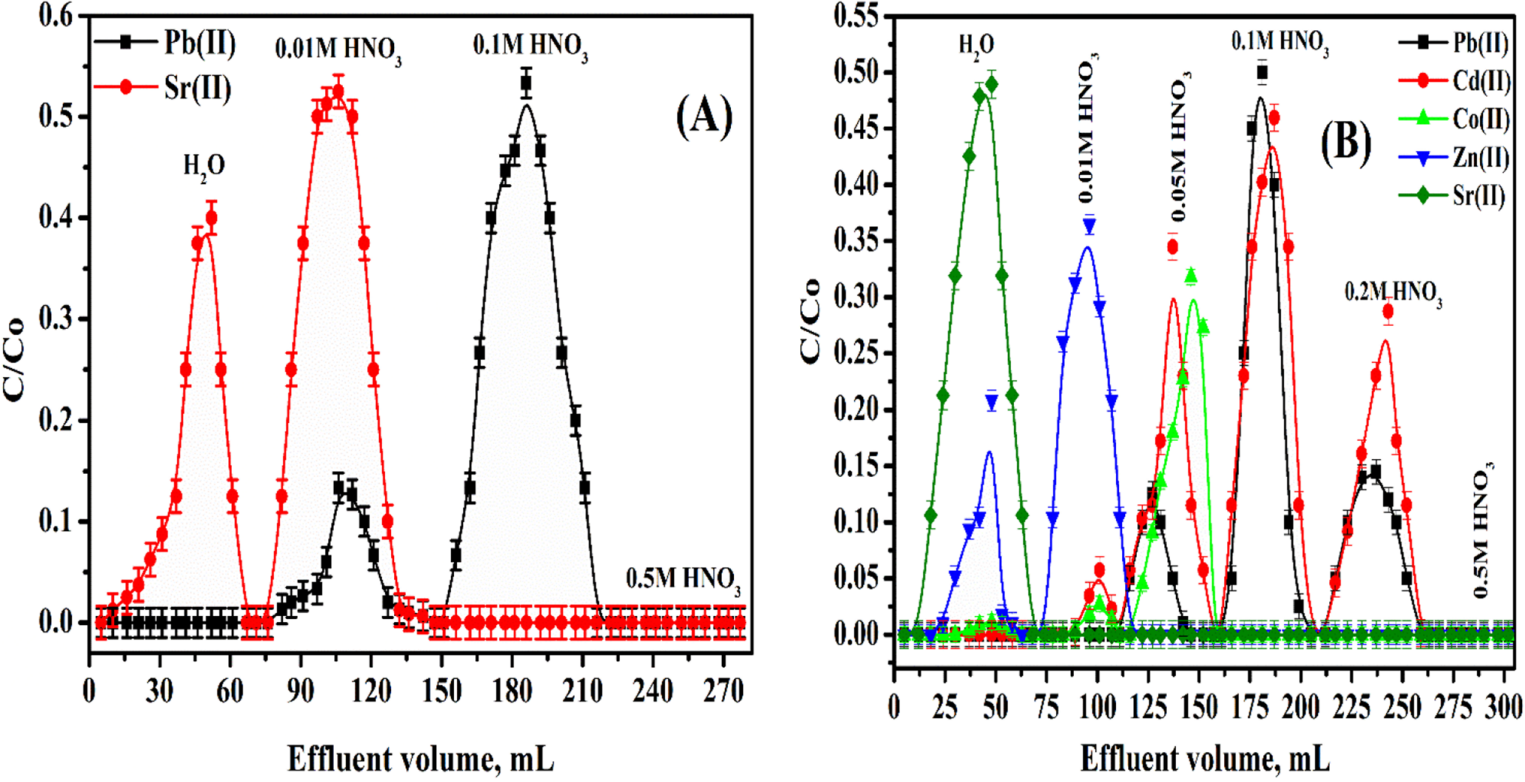
Chromatographic separation of (**A**) binary system Pb(II) and Sr(II) and (**B**) multi-component system Pb(II), Cd(II), Co(II), Zn(II), and Sr(II) sorbed onto P(AA-AN)-talc at pH 4

The elution results for Pb^2+^ and Sr^2+^ are illustrated in Fig. 11(A). The elution of the investigated ions is studied using DDW and dissimilar HNO_3_ concentrations (0.01, 0.1, and 0.5 M). Sr(II) ions were released using DDW and 0.01 M HNO_3_ as eluents. However, the separation of Pb(II) ions was released using 0.01 and 0.1 M HNO_3_ as eluents. By 0.5 M HNO_3_, the column packed with P(AA-AN)-talc becomes free from any sorbed metal ions and can be reused again for chromatographic separation.

### Elution of multi-component system

The elution data for Pb^2+^, Cd^2+^, Co^2+^, Zn^2+^, and Sr^2+^ are displayed in Fig. 11(B). The elution of the studied cations is investigated using DDW and different HNO_3_ concentrations (0.01, 0.05, 0.1, 0.2, and 0.5 M). Figure 11(B) shows that Sr(II) ions were released using DDW as eluent, Zn(II) ions were released using DDW and 0.01 M HNO_3_ as eluents, and Co(II) ions were released using 0.01 and 0.05 M HNO_3_ as eluents. However, Cd(II) and Pb(II) ions were released using 0.05, 0.1, and 0.2 M HNO_3_ as eluents by 0.5 M HNO_3_, and the column packed with P(AA-AN)-talc becomes free from any sorbed metal ions and can be recycled again for column separation.

## Conclusion

In this work, P(AA-AN)-talc was fabricated, characterized, and employed for batch sorption of Pb^2+^, Cd^2+^, Co^2+^, Zn^2+^, and Sr^2+^ from multi-component aqueous solutions. P(AA-AN)-talc was synthesized by gamma irradiation-induced preparation at 50 kGy as a new nanocomposite. The distribution coefficients at different pH have selectivity order: Pb^2+^  > Cd^2+^  > Co^2+^  > Zn^2+^  > Sr^2+^. The saturation capacity of the studied cations decreased by increasing the heating temperature. The sorption reaction is fast, and the reaction equilibrium is attained after 2 h. Pseudo-second-order kinetic is applicable with the sorption reaction mechanism. The sorption isotherm belongs to the Langmuir model, and the monolayer capacities of Pb^2+^, Cd^2+^, Co^2+^, Zn^2+^, and Sr^2+^ are 43.8, 37.9, 28.6, 23.6, and 9.09 mg/g, respectively. Thermodynamic parameters displayed that the ion exchange was endothermic and spontaneous. Column investigation reveals that P(AA-AN)-talc is more suitable for binary and multi-system. Finally, P(AA-AN)-talc can be worked as an efficient sorbent used for the separation of Pb^2+^ from Sr^2+^ (binary system) using 0.01 and 0.1 M HNO_3_. Also, P(AA-AN)-talc can be used as a promising sorbent for the separation of all studied cations from liquid waste utilizing DDW and different concentrations of HNO_3_ as eluents.

## Data Availability

Yes.
